
JOURNAL INFORMATION
==============================
NLM Title Abbreviation: J Med Radiat Sci
Journal ID: J Med Radiat Sci
NLM Journal ID: 101620352
Journal ID: JMRS

Journal of Medical Radiation Sciences

ISSN: 2051-3895
EISSN: 2051-3909
Publisher: Wiley

ARTICLE INFORMATION
==============================
PMCID: PMC11474810
DOI: 10.1002/jmrs.764
Article ID: JMRS764
Article version: 1
Subjects: Issue Information, Issue Information

Issue Information

Electronic publication date: 2024 Jun 14
Print publication date: 2024 Jun
Volume: 71
Issue: 2
First page: 165
Last page: 166
Copyright: © 2024 The Authors. Journal of Medical Radiation Sciences published by John Wiley & Sons Australia, Ltd on behalf of Australian Society of Medical Imaging and Radiation Therapy and New Zealand Institute of Medical Radiation Technology.
License: This is an open access article under the terms of the http://creativecommons.org/licenses/by/4.0/ License, which permits use, distribution and reproduction in any medium, provided the original work is properly cited.
License URL: https://creativecommons.org/licenses/by/4.0/

Figure count: 0
Table count: 0
Page count: 4

source-schema-version-number: 2.0
cover-date: June 2024
details-of-publishers-convertor: Converter:WILEY_ML3GV2_TO_JATSPMC version:6.4.9 mode:remove_FC converted:15.10.2024

==============================

Volume 71, Issue 2 June 2024

### Journal of Medical Radiation Sciences

Open Access

The impact of Mepitel on SGRT setup accuracy
Scoping review in angiographic software navigation
Automation and AI in RT treatment planning
The coroner's perspective

WILEY

Journal of

### Medical Radiation Sciences

Official Publication of the Australian Society of Medical Imaging and Radiation Therapy and New Zealand Institute of Medical Radiation Technology

**Editor-in-Chief**

Cherry Agustin, *The Crown Princess Mary Cancer Centre Westmead, and Blacktown Cancer and Haematology Centre Sydney, Australia (email: Cherry.Agustin@newcastle.edu.au)*

**Deputy Editors**

**Medical imaging**

Karen Dobeli, *Brisbane, Australia*
*Royal Brisbane and Women's Hospital*

**Radiation therapy**

Paul Kane, *Wellington, New Zealand*
*University of Otago*

**Associate Editors**

Linda Bell, *Sydney, Australia*
Elizabeth Brown, *Brisbane, Australia*
Jillian Clarke, *Sydney, Australia*
James Crowhurst, *Brisbane, Australia*
Karen Dobeli, *Brisbane, Australia*
Gay Dungey, *Wellington, New Zealand*
Rhys Fitzgerald, *Bundaberg, Australia*
James Hayes, *Christchurch, New Zealand*
Peter Kench, *Sydney, Australia*
Kellie Knight, *Florida, USA*
Stephen Knight, *Brisbane, Australia*
Michael Neep, *Brisbane, Australia*
Don Nocum, *Sydney, Australia*
Dean Paterson, *Wellington, New Zealand*
Robba Rai, *Sydney, Australia*
Tristan Reddan, *Brisbane, Australia*
Warren Reed, *Sydney, Australia*
Kelly Spuur, *Wagga Wagga, Australia*
Andrea Thompson, *Auckland, New Zealand*
Nick Woznitza, *London, United Kingdom*

**Biostatistics Editor**

James Stanley, *Wellington, New Zealand*

**Editorial Review Board**

Patrick Brennan, *Sydney, Australia*
Rob Davidson, *Canberra, Australia*
Georgia Halkett, *Perth, Australia*
Sarah Lewis, *Sydney, Australia*

**International Advisory Panel**

Kamarul Amin Abdullah, *Malaysia*
Nicole Harnett, *Toronto, Canada*
Michelle Leech, *Dublin, Ireland*
Paul Lockwood, *Canterbury, United Kingdom*
Eric Pei Ping Pang, *Singapore*
Wilfred CG Peh, *Singapore*
Suresh Rana, *Miami, USA*
Ronnie A Sebro, *Florida, USA*
Euclid Seeram, *Burnaby, Canada*
Vincent WC Wu, *Hong Kong SAR, China*
Michael Ying, *Hong Kong SAR, China*

**News and Online Editors**

Darien Montgomerie, *Wellington, New Zealand*
Adam Westerink, *Brisbane, Australia*

---

For submission instructions, subscriptions and all other information, visit wileyonlinelibrary.com/journal/jmrs

**Disclaimer:** The Publisher, Australian Society of Medical Imaging, New Zealand Institute of Medical Radiation Technology and Radiation Therapy and Editors cannot be held responsible for errors or any consequences arising from the use of information contained in this journal; the views and opinions expressed do not necessarily reflect those of the Publisher, Australian Society of Medical Imaging and Radiation Therapy or Editors, neither does the publication of advertisements constitute any endorsement by the Publisher, Australian Society of Medical Imaging and Radiation Therapy or Editors of the products advertised.

Journal of
Medical Radiation Sciences

**Volume 71, Issue 2** **June 2024**

### Contents

**Editorials**

The importance of quality management systems in nuclear medicine departments .................... 167
*Kunthi Pathmaraj*

Partnering with Consumers – Why would I do it? What is it? How do I do it? ............................ 170
*Jodie Nixon*

Artificial Intelligence and the future of radiotherapy planning: The Australian radiation therapists prepare to be ready ........................................................................................ 174
*Vanessa Panettieri, and Giovanna Gagliardi*

**Original Articles**

The impact of a prophylactic skin dressing on surface-guided patient positioning in chest wall Radiation Therapy ............................................................................................... 177
*James Cumming, Kenton Thompson, Katrina Woodford, Vanessa Panettieri, and Daniel Sapkaroski*

Quality audits of nuclear medicine practices in a middle-income African setting ........................ 186
*Magdalena Lutaka, Aladdin Speelman, Subhadranalene Naidoo, and Roswita Hamunyela*

Effect of using different strips on reducing the most common error in panoramic imaging: A randomised controlled trial on palatoglossal air space shadow ............................................... 194
*Andisheh Mokhtari, Sedigheh Razi, Kasra Rahimipour, and Tahmineh Razi*

A wellbeing podcast for diagnostic radiography students ........................................................... 203
*Emily Girard, Amanda Punch, and Yobelli Jimenez*

Barriers to radiographers' use of radiation safety principles: A qualitative perspective ................ 214
*Berit Møller Christensen, Anna Bjällmark, Irine Maghanwi Ndipen, Shilan Shamon Afram, and May Bazzi*

Perspective of radiography science – a document analysis of dissertations.................................. 222
*Sanna Törnroos, Helena Leino-Kilpi, Mervi Siekkinen, and Eija Metsälä*

Diagnostic value of colour Doppler ultrasound in differentiating malignant and benign nodules in thyroiditis background................................................................................ 233
*Seyed Ali Alamdaran, Melika Farshidianfar, Alireza Masoumi, Masoud Mahdavi Rashed, and Masoumeh Jaberi*

Estimation and comparison of the effective dose and lifetime attributable risk of thyroid cancer between males and females in routine head computed tomography scans: a multicentre study ........ 240
*Daryoush Khoramian, Mohammad Haghparast, Adnan Honardari, Ebrahim Nouri, Esmail Ranjbar, Razagh Abedi-Friouzjah, Shiva Zarifi, Choirul Anam, Milad Najafzadeh, and Mahdieh Afkhami-Ardakni*

Introducing fitting models for estimating age-specific dose and effective dose in paediatric patients undergoing head, chest and abdomen–pelvis imaging protocols: a patient study .......... 251
*Mohammad Reza Deevband, Habib Mohammadi, Yazdan Salimi, Ahmad Mostaar, Niloofar Deravi, Mobina Fathi, Kimia Vakili, Shirin Yaghoobpoor, Mehdi Ghorbani, Abolhasan Divband, and Meysam Tavakoli*

**Review Articles**

Exploring software navigation tools for liver tumour angiography: a scoping review ........ 261
*Nathan Brunskill, John Robinson, Don Nocum, and Warren Reed*

A systematic review of brain metastases from lung cancer using magnetic resonance neuroimaging: Clinical and technical aspects ........ 269
*Sadegh Ghaderi, Sana Mohammadi, Mahdi Mohammadi, Zahra Najafi Asli Pashaki, Mehrsa Heidari, Rahim Khatyal, and Rasa Zafari*

**Commentaries**

Automation and artificial intelligence in radiation therapy treatment planning ........ 290
*Scott Jones, Kenton Thompson, Brian Porter, Meegan Shepherd, Daniel Sapkaroski, Alexandra Grimshaw, and Catriona Hargrave*

See something, say something: the coroner's perspective ........ 299
*Kristal Lee, Kriscia Tapia, Mo'ayyad E. Suleiman, and Ernest Ekpo*

**How I do it**

Meaningful consumer engagement in medical radiation sciences: enhancing quality improvement and research projects ........ 304
*Amy Brown, Brianna McCoola, Yovanna Funnell, and Catriona Hargrave*

Framework for a radiography student podcast ........ 312
*Emily Girard, Amanda Punch, and Yobelli Jimenez*

**Continuing Professional Development**

Continuing Professional Development - Medical Imaging ........ 318
Continuing Professional Development - Radiation Therapy ........ 319

ISSN: 2051-3895

© 2024 *Journal of Medical Radiation Sciences published by John Wiley & Sons Australia, Ltd on behalf of Australian Society of Medical Imaging and Radiation Therapy and New Zealand Institute of Medical Radiation Technology*
